# Medico-Legal

**Published:** 1910-03

**Authors:** John A. Rafter


### PROGRESS IN MEDICAL SCIENCE.

### Medico-Legal

CONDUCTED by JOHN A. RAFTER, M. D.

AT the October meeting of the medico-legal section of the Cleveland Academy of Medicine which was held October 29, 1909, two notable papers were read on a medico-legal subject, each being entitled "Tests of Insanity in Civil Courts." One was presented by Judge Benjamin A. Gage, a well known lawyer, the other by Dr. W. B. Laffer, a leading and capable physician. The papers are too long to be republished entire, but each contains so much that will interest any physician who is likely to be called into court to give an opinion on the subject of which the papers treat, that liberal extracts are reproduced in the JOURNAL. Credit is given the *Cleveland Medical Journal,* December, 1909:

---

The criterion of insanity in the civil court varies from that in the criminal and probate court. In the different actions in the civil court alone the law recognises various degrees of mental derangement and responsibility that are hardly scientific from an alienist's viewpoint. So in considering the possible cause for the individual's insanity we must bear in mind that alcohol seems to produce about 20 per cent. of the insanities and inherited predisposition to insanity is found in from 60 to 90 per cent. of the cases.

---

"Substantially every individual at some time or other during his life is exposed, in many cases repeatedly, to many of the so-called exciting causes of insanity, both mental and physical, and yet despite this fact we find that sanity is the rule, and insanity the exception." A cause may be sufficient to cause insanity in one and not in another, or the cause may be sufficient only at times with the same individual. Yet physicians are usually called upon to investigate the responsibility of the individuals with the legal restrictions according to the action in question.

---

The test in all cases in the civil courts in which insanity is involved as an issue, seems to be whether or not the party was mentally responsible for and appreciated the nature and effect and consequence of the *particular act* in question, for it is perfectly possible for a monomaniac to be held irresponsible civilly in an action based upon some act within the scope of his particular mania, and to be held liable civilly in an action based upon an act outside of that scope. Or expressed otherwise, insanity as a foundation for relief or as a defense must exist with reference to and concerning the *particular act involved.*

*Mere weakness of mind alone* is not insanity, and the courts will not undertake to interfere in every case in which a superior or more astute intellect has obtained an advantage over a more feeble mind.

---

The weight of authority seems not to require so high a standard of mental vigor and power for the execution of a valid will as is required for the execution of deeds, mortgages or ordinary contracts, and the generally accepted test of testamentary capacity in this State is whether at the time of the execution of the will the testator possessed an understanding of the nature of the business in which he was engaged, a recollection of the property he meant to dispose of, a remembrance of the persons having claim upon his bounty and the manner in which it was to be distributed.

---

A contract, written or oral, of necessity, involves two or more parties, and it is a requirement of the law that voluntary and intelligent assent to its terms and conditions be given by each of such parties. Hence, if either party at the time the agreement was entered into, was of unsound mind or insane in a degree recognised by the law, as hereinafter defined, such insanity may constitute the foundation of an action by that party. for affirmative relief, such as rescission or annulment of the agreement; or it may serve as a defense against its enforcement, subject to various rules of law, which it is needless to discuss here.

---

Insanity may be pleaded, as a defense against the enforcement of some remedy permissible against the sane, as in an action to enforce specific performance of an agreement to convey real estate. In such case, if it be proved that the defendant at the time of the execution of the agreement was insane, a decree of specific performance will be refused. If a lunatic destroys property he is responsible in damages for the value thereof, regardless of the fact that he was not capable of having any evil intent, and his insanity would be no defense. If he were prosecuted criminally however for malicious destruction of property, malicious intent would be a necessary ingredient of the offence and his insanity would be a complete defense thereto.

It is not possible to set forth the varying expressions employed by courts in laying down rules for the determination of sanity in each of the various cases which have been considered. It has been held, however, that when the validity of a conveyance has been attacked, the grantor is usually held sane, for the purpose of making such conveyance "if he has sufficient capacity fully to comprehend the nature and effect of his act." And with reference to contracts in general, it is said that it is essential to their validity that the parties have capacity to understand and agree, and that to avoid the contract they must lack sufficient mind to understand in a reasonable manner the effect of the act in which they are engaged. The proof of insanity must go to this length, in order to render the contract void.

It is also held that "either the absence of intellect or a great mental aberration is sufficient to render a contract void." When unsoundness of mind is relied upon as a defense to an action upon a contract, or as a basis of relief upon it, that unsoundness or insanity must be proved to be of such character as that the person had no reasonable perception or understanding of the nature and terms of the agreement.

---

In case of the validity of a will, the law requires the testator to have a "sound disposing mind" either at the time when he gave instructions for the will to be prepared or at the actual moment of its execution: it is not necessary that he should have a "sound disposing mind" on both occasions. It often falls to the lot of a medical man to examine a patient in order to decide whether he is of a sound disposing mind. One should, then, endeavor to ascertain whether the individual is capable of enumerating on the one hand the details of his estate and on the other the individuals who have any reasonable claim to benefit from it and whether there appears to be any person who has exercised undue influence on his decisions.

Undue influence must be such as to destroy the free agency of the testator and it is immaterial how little the influence was if free agency is thereby destroyed. The physician has a difficult task in determining this, which is done by investigating the mental habit of the testator, the contents of the will, the amount of his property and the personal and blood relationship of the beneficiaries to the testator.

---

One should ascertain whether the testator is suffering from any delusion which might influence his decision and whether he has any insane or unnatural dislike or suspicion of any members of his family, who would in the ordinary course become beneficiaries; if a delusion does not influence the mind of the testator in making the will it cannot affect its validity.

Also, whether, having once announced his decisions, he is capable of recapitulating them, say a few days later. The law upholds a will made from eccentric, frivolous or capricious motives, provided it can be shown that the will represents the true wishes of the testator and was not the result of an eccentricity, frivolity, or caprice of the moment.

---

Most wills are made after the individual has reached an age when the presence of senile degenerative changes, or the more acute lesions resulting from these changes have warned the testator or his friends that his expectancy of life is not great. In senile dements the delusions often relate to property and they believe that they are being defrauded and this leads them to lose confidence in their former trusted relatives and to intrust or will their property to designing persons who talk plausibly and seem to do as desired. The loss of natural affection for relatives is one of the commonest symptoms of insanity, and may cause the testator to leave out his relatives from his will.

Inebriety may affect the validity of wills because the inebriate may not possess testamentary capacity or because the influence of insane delusions may be so pronounced that the will cannot be supported, or because the evidence of undue influence is so apparent that it cannot be disregarded. At the time of intoxication or by reason of habitual inebriety or alcoholic insanity the mind of the testator may be so confused, enfeebled or diseased that he cannot fulfil the requirements of the law in regard to his testamentary mental capacity. While on the other hand he may be suffering from any of these forms of inebriety, and yet execute a will that can very properly be sustained at law, while medically we cannot grant that the testator is responsible with a mind acting normally.

---

Now in the case of contracts, the occurrence of insanity does not excuse the party from the performance of a contract made previously to his becoming insane. Insanity in the case of contracts must be to such a degree that there is entire absence of intellect, or at least great mental aberration, so that there is an inability to understand the contract or to properly consent to the same. Temporary or even permanent monomania or mere mental weakness is not a sufficient cause to set aside a contract.

---

Marriage is a contract the validity of which, as in other contracts, depends upon the consent of the parties and the agreement of their minds. A physician is often called upon to decide whether at the time of the marriage the plaintiff's mind was so affected by an alcoholic intoxication or alcoholic degeneration of the brain, or an insanity, as to be incapable of understanding the meaning of the act. Cases of incapacity in matrimonial actions are often confused with and hard to distinguish from cases of fraud, mental duress and mental weakness, due to age, sickness, action of drugs, and particularly undue influence. All of which may be cause for such suits. The feeblemindedness or insanity must be such as to affect an understanding of the marriage contract. Persons affected with monomania, pyromania, kleptomania or dipsomania may still be legally fit to marry. That which would disqualify a person from entering into *property* relations might not invalidate a marriage.

---

Physicians may be called upon to give expert testimony in regard to responsibility in suits for breach of promise when the defendant claims he was mentally incompetent at the time the promise was given. A promise to marry made during an attack of dipsomania in chronic alcoholism, in alcoholic or other insanity, may be avoided if the defendant can show that at the time of the promise he had not sufficient mind to make a valid contract or that his mental faculties were so perverted that he did not comprehend the nature of the act.

---

Most insurance policies have a suicide clause and a physician is often asked to determine the mental condition of a suicide. The suicide of the insured is not a breach of warranty in the application that he will not "die by his own hand" if at the time of the suicide the reasoning faculties were so far impaired that insanity may constitute the foundation of an action by that party the insured was unable to understand the moral character, general nature, consequences and effect of the act, or when he was impelled thereto by an irresistible insane impulse.

---

Malicious deeds of the insane, such as breaking or destroying objects of greater or less value either planning or permitting to have the suspicion and blame fall on others are not uncommon.

Insanity is no excuse for a civil wrong not involving as an ingredient a specific intent or malice, and the injured party is entitled to damages on the principle that every man is entitled to possess inviolate his personal security, liberty and reputation The law, in spirit at least, recognises attenuated responsibility; the degree of which is largely based on the medical man's opinion as to the amount of alienation the defendant has. The jury will not likely award heavy damages, say in a case of slander in which the offender is known to be so insane that nobody would attach any importance to his statements.

---

Physicians may be called upon in an action for damages in which it is claimed an injury was the immediate or remote cause of insanity. We should remember that traumatic insanity is uncommon. It may be immediate but, as a rule, does not arise until long afterwards and a distinct change in character being noticed in the interval. Traumatic insanity may result from slight damage or severe injury. Traumatism may be followed by organic changes. Head injuries may affect the brain by fracture of the skull, depression of bone, by laceration and hemorrhage, and by simple concussion; there may be only a capillary hemorrhage. Traumatic insanity can occur in those not predisposed by heredity, alcoholism, syphilis, sexual excesses, worry and overwork, but is more common in the predisposed.

The so-called confusional insanity is the type most often seen in these cases, especially when following shock, fright or exhaustion.

---

We all know that there may be prenatal, natal or postnatal injuries to the child's brain that will result in idiocy, imbecility, weakmindedness, epilepsy, or paralysis for which the obstetrician or child's caretakers may be blamed. If convulsions follow cranial injuries they are apt to persist and the child may present the progressive mental failure of the epileptic. Head injuries in youth may produce conditions such as seen in children, also maniacal outbreaks, morbid impulses, perversion of character and the so-called moral insanity and monomania.

Everybody knows insane people with whom one may talk for a long time without noticing anything out of the way, other than a certain queerness or slight degree of eccentricity up to the time when they are led to say that, "they are emperors of the wilderness, or martyrs living in huts." Patients with the so-called lucid mania have, on superficial examination, every appearance of reason. A person may reason well and yet be insane, the premise being wrong and founded on delusions. This is frequently seen in paranoia.

---

Medical evidence constitutes an important part of all personal-injury actions, both in criminal and civil practice. In criminal actions the guilt of the defendant cannot be established without medical evidence showing a connection between the means used by the defendant and the crime charged. In the large number of civil actions claiming damages for injury to the person, proof of the defendant's negligence must be followed by medical evidence showing that the injuries and disabilities are the result of that negligence.

---

In New York and Kings counties, negligence actions constitute about 40 per cent. of all actions on the Supreme Court calendar. If to these negligent actions be added actions arising from life and health insurance, and from mental capacity in the management of one's estate, as well as the ability to dispose of an estate by will, it will be seen that medical evidence may be required in about one-half of civil law practice.

---

